# The effectiveness of educational, behavioural, and cognitive self-management support interventions for chronic migraine: a systematic review

**DOI:** 10.1017/S1463423625100571

**Published:** 2025-12-03

**Authors:** Aiva Hailston, Natasha Davies, Mariam Ratna, Manjit Matharu, Martin Underwood

**Affiliations:** 1 Warwick Clinical Trials Unit, Warwick Medical School, University of Warwick, Coventry, UK; 2 Headache and Facial Pain Group, University College London, London, UK; 3 Headache and Facial Pain Group, National Hospital for Neurology and Neurosurgery, London, UK; 4 University Hospitals Coventry and Warwickshire, Coventry, UK

**Keywords:** Chronic migraine, headache, migraine, non-pharmacological interventions, self-help, systematic review

## Abstract

**Aim:**

In this systematic review, we identify and critically appraise randomised controlled trials of effectiveness of available educational, behavioural, cognitive, and self-management support interventions for individuals with chronic migraine.

**Background:**

Non-pharmacological interventions have the potential to help people living with chronic migraine. Little is known about their true effectiveness.

**Methods:**

We searched Cochrane, Embase, Medline, PsychINFO, Scopus, and Web of Science for randomised controlled trials assessing the effectiveness of educational, behavioural, cognitive, and self-management support interventions, compared to usual care, for adults with chronic migraine. Our outcomes of interest were headache frequency, headache-related disability, quality of life, pain intensity, medication consumption, and psychological wellbeing at baseline and follow-up.

**Findings:**

We included six randomised controlled trials (713 participants) whose interventions met our inclusion criteria: two educational, two psycho-educational, and two behavioural interventions. Trial heterogeneity precluded statistical pooling. Several small trials reported some between-group differences. One trial (*N* = 177) found more people had ≥50 reduction in headache frequency at 12 months following a psychological (mindfulness-based) intervention added to acute medication withdrawal in people with medication overuse headache: 43/89 (48%) control vs. 69/88 (78%) intervention, *p* < 0.001. However, the largest included study (*N* = 396) had effectively excluded the possibility that their intervention had a worthwhile effect on headache-related disability at 12 months; mean difference in Headache Impact Test (HIT-6) 0.7 (95% Confidence Interval −0.65 to 1.97). Current evidence does not support the use of educational, behavioural, cognitive, and self-management support interventions for individuals with chronic migraine to improve headache-related symptoms and quality of life. Very limited evidence suggests they may contribute towards headache frequency reduction.

## Introduction

Migraine is the second largest cause of years lived with disability and the 14^th^ leading cause of disability-adjusted life years globally (Steiner *et al.*, 2020). Chronic migraine, defined as headaches occurring on at least 15 days a month, with eight of them having migraine features for more than three months (Olesen, 2018), results in substantially greater disability than episodic migraine (Stokes *et al.*, 2011). It affects relationships, careers, finances, and general health (Buse *et al.*, 2019). Over half (57%) of people with chronic migraine miss five or more days of household activities over a three-month period (Bigal *et al.*, 2008). People with chronic migraine access more medical resources than people with episodic migraine, with healthcare costs being nearly five times greater (Negro *et al.*, 2019). Topiramate, onabotulinumtoxin A (Botox), and calcitonin gene-related peptide (CGRP) monoclonal antibodies (MAbs) are of proven effectiveness for reducing headache/migraine days and, except for topiramate, improving headache-related quality of life for people with chronic migraine (Naghdi *et al.*, 2023). The effect sizes are, however, modest, typically no more than 2.0 – 2.5 headache/migraine days per month. This means that even with adequate preventive drug treatment many people continue living with chronic migraine. In common with other chronic pain disorders there may be a role for educational, behavioural, cognitive, and self-management support interventions for people with chronic migraine (Probyn *et al.*, 2017). Their role, if any, has not yet been established. A 2019 Cochrane review of psychological therapies for prevention of migraine, episodic or chronic, concluded that it was not possible to determine if psychological interventions were effective for migraine because of the absence of high-quality research (Sharpe *et al.*, 2019).

## Method

### Study registration

We wrote the protocol in May 2021 and prospectively registered it with the International Prospective Register of Systematic Reviews (CRD42021260376). We followed the Preferred Reporting Items for Systematic Reviews and Meta-Analyses (PRISMA) guidelines for reporting the review.

We used the PICO framework to guide the development of the research question. Population: adults meeting the International Classification of Headache Disorders, 3rd edition (ICHD-3) criteria for chronic migraine, with or without aura. Interventions: educational, behavioural, and cognitive self-management support interventions. Control: usual therapy, typically involving pharmacological management. Outcomes: headache frequency, headache-related disability, pain intensity or severity of symptoms, quality of life, psychological wellbeing, and medication consumption.

### Search

We developed search strategies (Supplementary Material 1) with expert librarian support to retrieve peer-reviewed randomised controlled trials (RCT), comparing educational, behavioural, cognitive, and self-management support interventions against usual care or sham interventions. We used Medical Subject Headings (MeSH) and free text searching, considering alternative spellings and truncations. As criteria for ‘chronic migraine’ were first introduced in 2004. We searched Cochrane, Embase, Medline, PsychINFO, Scopus, and Web of Science databases for studies published between 2004 and 18th November 2021. We updated this on the 27^th^ of July 2023. We also carried out backward citation tracking. We retrieved full-text articles published in English language in peer-reviewed scientific journals, including electronic versions. We excluded grey literature and conference articles.

### Eligibility criteria

#### Inclusion criteria

- Adults ≥ 18 years old
- International Classification of Headache Disorders-3 (ICHD-3) definition of chronic migraine with or without aura, either stated by authors or evident from the recruited population
- Studies with mixed types of headaches, with at least 75% of the participants with chronic migraine
- Chronic migraine with or without medication overuse
- Behavioural, cognitive, educational, self-management support interventions
- Behavioural interventions with an exercise component

#### Exclusion criteria

- Headache due to secondary causes
- Headaches <15 days a month
- Headaches with no features of migraine (National Institute for Health and Care Excellence, 2021)
- Exercise only trials
- Interventions involving use of any apparatus, i.e., acupuncture, transcutaneous electrical stimulation
- Sham interventions
- Trials with <15 participants per treatment arm

### Selection process and data extraction

Two reviewers (AA, ND) independently screened all records by title and abstract, followed by full-text analysis of the selected trials. We discussed our decisions. A third reviewer (MU) acted as an arbitrator. We contacted authors for clarification where there was ambiguity about whether the trial met the eligibility criteria. The excluded articles following full-text appraisal are listed in Supplementary Material 2.

For each included study, AA and ND independently extracted data on country, size, intervention type, duration, delivery mode, materials for data collection, follow up, and declaration of interest.

### Risk of bias assessment

AA and ND independently assessed the risk of bias, as per the Cochrane Risk of Bias Tool v2.0 (Sterne *et al.*, 2019), with MU reviewing any disagreements. For one trial, for which MU is the chief investigator, an independent reviewer (JB) considered any disagreements. We classified the papers as having low, some concerns, or high risk of bias, as per the Cochrane Handbook for Systematic Reviews (Higgins *et al.*, 2019).

### Data synthesis

We classified the studies based on how the authors described the interventions – psychological, behavioural, or educational interventions. We used the general framework for synthesis outlined in section 9.2A in the Cochrane Handbook for Systematic Reviews of Interventions (Higgins *et al.*, 2019). We extracted mean, standard deviation, confidence intervals, effect size, and p-values for treatment and control groups for the following outcomes: headache frequency, pain intensity/severity of symptoms, quality of life, psychological wellbeing, and medication consumption. We compared the values at baseline and at follow-up. We assessed whether the data were appropriate for statistical pooling for meta-analysis and produced a narrative synthesis of the results when pooling was not feasible.

### Deviations from protocol

In error, we omitted headache-related disability as a specified outcome from our original protocol. This has been identified as being of similar importance to people with chronic migraine as headache/migraine days (Haywood *et al.*, 2021). We have therefore also extracted and presented these data.

## Results

**Figure 1.**
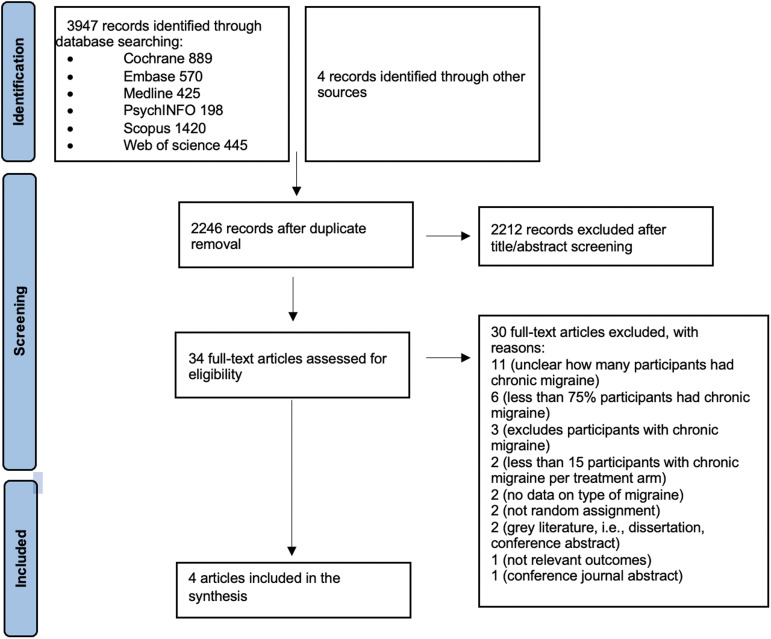
Study flow diagram November 2021.

In November 2021, we identified 3947 articles through searching databases and four trials following backward citation searching. After screening, we assessed 34 full-text articles and included four studies (Figure 1).

**Figure 2.**
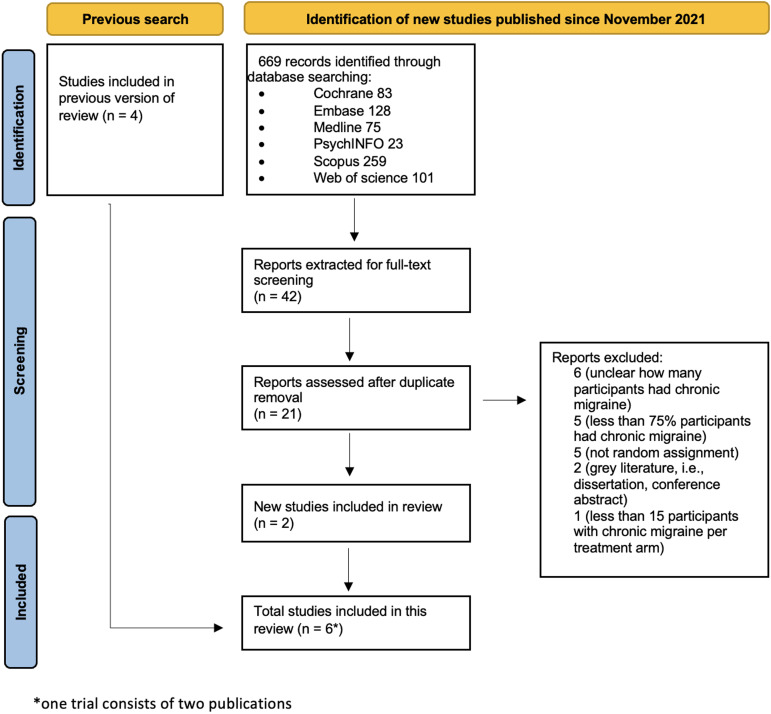
Study flow diagram July 2023.

We updated our search in July 2023 and found two eligible studies published since November 2021 (Figure 2). One study (Grazzi *et al.*, 2023, 2022) was published as two separate publications, with three-month and 12-month follow up.

Three studies were done in the USA (Calhoun and Ford, 2007a; Seng *et al.*, 2021; Smitherman *et al.*, 2016), and one each in Iran (Rashid-Tavalai *et al*., 2015), Italy (Grazzi *et al.*, 2023, 2022), and the UK (Underwood *et al.*, 2023a, 2023b). Two studies (Seng *et al.*, 2021; Underwood *et al.*, 2023a) included mixed populations of episodic and chronic migraine and reported some results separately just for those people with chronic migraine. The six trials included 713 participants with chronic migraine, with sample sizes ranging from 31 (Smitherman *et al.*, 2016; Seng *et al.*, 2021) to 396 (Underwood *et al.*, 2023a). No two studies used similar interventions: Calhoun and Ford (2007a), behavioural sleep modification; Grazzi *et al.*, (2023, 2022), mindfulness and education about migraine as an addition to medication withdrawal in people with medication overuse headache; Rashid-Tavalai *et al*. (2015), coping skills training; Seng *et al.* (2021) mindfulness-based cognitive therapy; Smitherman *et al.* (2016), cognitive-behavioural therapy for insomnia (CBTi); Underwood *et al.*, (2023a), an educational and self-management support intervention. Two studies (Calhoun and Ford, 2007a; Smitherman *et al.*, 2016) used sham interventions for their control group. The rest used usual care controls. All studies recruited participants from physician referrals or clinic attendance; additionally, Seng *et al.* (2021) used advertisements. Underwood *et al.* (2023a) allowed participants to self-refer; however, most participants were recruited by writing to them.

The participants’ mean age ranged from 30 to 47 years, and they were predominantly female (83%–100%). Underwood *et al.* (2023a), Seng *et al.* (2021), and Smitherman *et al.* (2016) reported that their population was predominantly white (80%, 77%, and 81% respectively). No other studies reported data on ethnicity. There were 40% participants with a formal diagnosis of depression in the study by Calhoun and Ford (2007a), whereas all participants in the study by Smitherman *et al.* (2016) had insomnia. Underwood *et al.* (2023a) reported that of all participants, including those with chronic tension-type headache and episodic migraine 53% had probable anxiety and 22% had probable depression. Medication overuse headache was present in 100% participants in Grazzi *et al.* (2023, 2022), 74% in Calhoun and Ford (2007), 56% in Underwood (data on all participants (Underwood *et al.*, 2023a)), and 0% in Smitherman *et al.* (2016). Neither Rashid-Tavalai *et al*. (2015) or Seng *et al.* (2021) reported proportion with medication overuse headache.

We found protocols on clinicaltrials.gov for four studies (Grazzi *et al.*, 2023, 2022; Seng *et al.*, 2021; Smitherman *et al.*, 2016; Underwood *et al.*, 2023b). The remaining two studies (Calhoun and Ford, 2007; Rashid-Tavalai *et al*., 2015) did not refer to a protocol.

### Risk of bias

**Figure 3.**
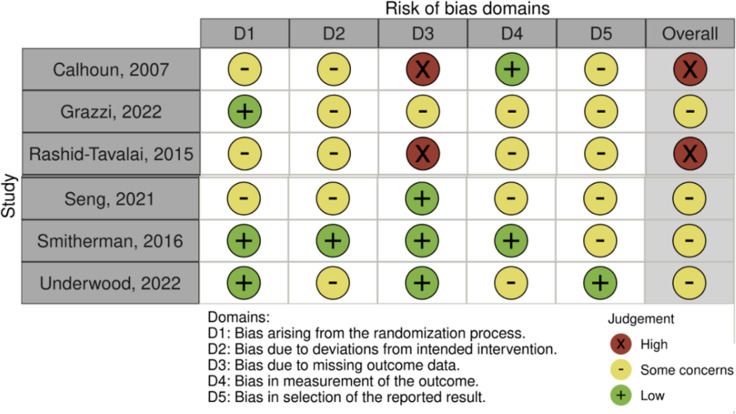
Risk of bias assessment of the included studies.

We judged two trials to be at high risk of bias (Calhoun and Ford, 2007; Rashid-Tavalai *et al*., 2015) and four had some concerns (Grazzi *et al.*, 2023, 2022; Seng *et al.*, 2021; Smitherman *et al.*, 2016; Underwood *et al.*, 2023a) (Figure 3, Supplementary material 3).

The heterogenous nature of the interventions tested and outcome reporting mean that statistical pooling of results was not appropriate. Both Underwood *et al.* (2023a) and Grazzi *et al.* (2023, 2022) measured HIT-6 at 12 months. However, the different analyses presented mean data pooling was not possible. We therefore present a narrative summary. No studies reported separately on migraine days (Table 1). For reporting purposes, we have categorised the studies broadly as educational interventions (Rashid-Tavalai *et al*., 2015; Underwood *et al.*, 2023a), psychological interventions (Grazzi *et al.*, 2023, 2022; Seng *et al.*, 2021) and behavioural interventions (Calhoun and Ford, 2007; Smitherman *et al.*, 2016). We based this on authors’ descriptions in the original studies.

**Table 1.** Characteristics of included studies

Study	Size	Intervention type	Control	Duration	Delivery	Assessment at	Materials for data collection	Primary outcomes	% of individuals with CM + Medication overuse Headache
Calhoun and Ford, 2007	*N* = 43 Intervention group *N* = 23 Control group *N* = 20	Behavioural sleep modification (BSM)	Sham Control (placebo behavioural activities) + usual care	5 × 20-min sessions over 12 weeks	in-person	6 and 12 weeks	1. Headache diary	1.Headache frequency 2.Pain intensity	74%
Grazzi, 2022 & 2023	*N* = 177 Intervention group N = 88 Control group *N* = 89	Psychoeducational (education about migraine + mindfulness)	Usual care	6 × 90-min weekly sessions over 6 weeks + 7-10 min home practice sessions	In-person	Three months & 12 months	1. Headache diary 2. Migraine-specific QoL questionnaire 3. MIDAS 4. WHODAS-12 5. Headache impact Test-6 (HIT-6) 6. Beck Depression Inventory – II; STAI-Y 7. Mindful attention and awareness scale (MAAS)	1. Headache frequency 2.Acute medication consumption 3. Quality of life 4. Headache-related disability 5. Anxiety and depression 6. Symptomatic medication intake 7. Self-awareness	100% (all participants had chronic migraine + medication-overuse headache)
Rashid-Tavalai *et al.,* 2015	*N* = 35 Intervention group *N* = 18 Control group *N* = 17	Educational	Usual care	7 × 2h weekly sessions over 7 weeks	in-person	7 weeks	1. WHO-QOL questionnaire 2. General self-efficacy scale 3. Ways of coping questionnaire 4. Migraine headache index	1. Pain intensity 2. Self-efficacy 3. Quality of life 4. Emotional-oriented strategies 5. Problem-oriented strategies	Not stated
Seng, 2021	*N* = 31 Intervention group *N* = 15 Control group *N* = 16	Psychoeducational (mindfulness-based CBT)	Waiting list or Usual care	8 × 75min weekly sessions over 8–10 weeks	in-person (up to 3/8 sessions could be delivered over the phone)	16 weeks	1. MIDAS 2. Henry Ford Hospital Headache Disability Inventory 3. Daily headache diary 4. Migraine Disability Index	Headache-related disability	Not stated
Smitherman, 2016	*N* = 31 Intervention group *N* = 16 Control group *N* = 15	Behavioural CBT for insomnia (CBTi)	Sham Control (behavioural activities on ‘Lifestyle Modification’)	3 × 30min bi-weekly sessions, with 2 weeks between each session	in-person	2 and 6 weeks	1. Actiwatch II (actigraphy) 2. Headache and sleep diary 3. MIDAS 4. Headache impact Test-6 (HIT-6) 5. Pittsburg Sleep Quality Index (PSQI) 6. Epworth Sleepiness Scale (ESS) 7. PHQ-9 8. Generalised Anxiety Disorder 7-item Scale (GAD-7) 9. Credibility/Expectancy Questionnaire (CEQ) 10. Participant Adherence rating scale	Headache frequency	Excluded participants with medication overuse headache
Underwood et al., 2023a	*N* = 396 Intervention group *N* = 205 Control group *N* = 191	Educational and self-management	Usual care and relaxation materials (CD)	2 × one-day group sessions one week apart, followed by one-to-one nurse interview and telephone support	In-person with additional telephone support	12 months	1. Headache impact Test-6 (HIT-6) 2. Chronic Headache Quality of Life Questionnaire 3. Hospital Anxiety and Depression Scale 4. Pain Self-Efficacy Questionnaire 5. Social Activity: Social Integration Subscale of the Health Education Impact 6. Diary records/smartphone app for headache characteristics	Headache-related quality of life	56% of all trial participants including those with chronic tension type headache and episodic migraine

### Headache frequency

#### Psychological interventions

The results of all the studies are summarised in Table 2. At three months, Grazzi *et al.* (2023, 2022) reported a difference in headache frequency; the control group had 31.3 (SD 20.3) headache days, and the intervention group had 26.6 (SD 21.8). They found a statistically significant time by group interaction (*p* = 0.029). They also report that 45/83 (54%) of control group and 60/79 (76%) of intervention had ≥50% reduction in headache frequency (p=0.004). At 12 months, there was a statistically significant reduction in the headache frequency in the intervention group when compared to the control group (*p* < 0.0001); 69/88 (78%) of individuals in the intervention group achieved a 50% or more headache frequency reduction, compared to 43/89 (48%) in the control group.

**Table 2.** Summary of results; NS = not significant; * = significant at *p* = .05, **significant at *p* = .025, ‘-‘ = data not reported

Study ID	Measures used (scale range if available)	Follow up time	Intervention			Control			Effect estimate	95% CI	p-value
			*N*	Baseline mean (SD)	Follow up mean (SD)	*N*	Baseline mean (SD)	Follow up mean (SD)			
**Headache frequency**											
Calhoun and Ford, 2007	Number of headaches per 28 days	6 weeks	23	24.2 (−)	17.4 (−)	20	23.2 (−)	23.9 (−)	–	–	0.001*
Grazzi, 2022, 2023	Number of headache days per three months	3 months	88	66.3 (15.9)	26.6 (21.8)	89	63.1 (15.0)	31.3 (20.3)	–	–	0.025*
		12 months	76	–	–	78			–	–	<0.0001*
Seng et al, 2021	Number of headache days per 30 days	4 months	16	–	–	15	–	–	0.18	−4.63, 4.99	NS
Smitherman et al, 2016	Extrapolated Headache frequency (headache probability × 30)	2 Weeks	16	22.7 (−)	16.6 (−)	15	19.6 (−)	12.5 (−)	–	0.52, 2.15	0.883
		6 weeks			11.6 (−)			14.7 (−)	–	0.17, 0.91	.028**
**Headache-related disability**											
Grazzi, 2023	HIT-6 (36–78) ≥6 point improvement	12 months	76	–	–	78	–	–	2.45	−4.69, 1.28	NS
Seng et al, 2021	MIDAS (0–270)	4 months	16	- (−)	- (−)	15	- (−)	- (−)	−0.7 (favouring intervention)	−1.27, −0.08	<0.05*
	Henry Ford Hospital Headache Disability Inventory			- (−)	- (−)		- (−)	- (−)	−12.12	−25.17, 0.93	NS
Smitherman et al, 2016	MIDAS (0–270)	2 weeks	16	59.9 (39.0)	44.2 (43.1)	15	54.5 (41)	41 (46.2)	–	–	NS
		6 weeks			31.9 (33.2)			34.7 (34.5)	–	–	NS
	HIT-6 (36–78)	2 weeks		66.9 (3.8)	62.6 (5.3)		64.8 (3.9)	64.8 (3.9)	–	–	NS
		6 weeks			59.9 (5.5)			61.4 (8)	–	–	NS
Underwood et al, 2023a	HIT-6 (36–78)	12 months	159	–	61.4 (6.8)	149	–	62.7 (6.1)	−0.7	−1.97, 0.65	.325
**Pain intensity/severity of symptoms**											
Calhoun and Ford, 2007	Headache index (number of headaches per 28 days * headache severity, range 0–114)	6 weeks	23	46.7 (−)	28.3 (−)	20	50.2 (−)	44.1 (−)	–	–	0.01*
Rashid-Tavalai *et al.,* 2015	Migraine headache index (scale range not available)	7 weeks	18	8.17(2.13)	6.78 (2.34)	17	7.47 (2.03)	6.71 (2.77)	–	–	0.26
Seng et al, 2021	Average attack intensity/30 days (0–3)	4 months	16	–	–	15	–	–	−0.02	−0.16, 0.13	NS
Smitherman et al, 2016	Headache Severity (0–10)	2 weeks	16	5.2 (0.59)	5.1 (1.4)	15	5.4 (1.6)	5.2 (1.6)	–	–	–
		6 weeks			4.5 (1.5)			5.1 (2.1)	–	–	–
**Quality of life**											
Rashid-Tavalai *et al.,* 2015	World Health Organisation Quality of Life Questionnaire (16–112)	7 weeks	18	56.83 (9.86)	61.72 (10.85)	17	57.12 (10.69)	56.94 (9.61)	–	–	0.049
**Psychological wellbeing**											
Smitherman et al, 2016	PHQ-9 (0–27)	2 weeks	16	12.1 (5.8)	6.9 (4.8)	15	10.5 (4.5)	8.4 (4.7)	–	–	–
		6 weeks			6.3 (4.6)			8.6 (4.7)	–	–	0.054
	GAD-7 (0–21)	2 weeks		10.6 (6.4)	6.6 (5.2)		9.8 (5.3)	7 (4.6)	–	–	–
		6 weeks			6.3 (4.8)			6.9 (4.9)	–	–	0.430
**Headache-related disability responder analysis**											
**Study ID**	**Measures used (scale range if available)**	**Follow up time**	**Control** **n/N (%)** **≥6 point improvement**			**Intervention** **n/N (%)** **≥6 point improvement**			**Odds ratio**	**95% CI**	**p** **Chi** ^**2**^
Grazzi, 2023	HIT-6 (36–78)	3 months	50/79 (63%)			36/83 (43%)			2.25	1.20, 4.23	0.011
		6 months	47/76 (62%)			33/81 (41%)			2.36	1.24, 4.47	0.0008
		12 months	45/76 (59%)			29/78 (37%)			2.45	1.28, 4.69	0.006

Seng *et al.* (2021) did not find any effect of mindfulness-based cognitive therapy on headache frequency at four months. Mean difference in headache days/30 days was 0.18 (95% CI −4.63, 4.99).

#### Behavioural interventions

Calhoun and Ford (2007) reported a statistically significant reduction in mean headache frequency at six weeks, following behavioural sleep modification training: 17.4 vs 23.9 headache days in the previous 28 days (*p* = 0.01). Furthermore, none of the participants in the control group reverted to episodic migraine (*p* = 0.029) at follow-up.

Smitherman *et al.* (2016) reported that at two weeks post-intervention, the control group showed a greater reduction in headache frequency than the CBTi (Cognitive Behavioural Therapy intervention) group (36% and 27% respectively). The odds ratio of a participant having a headache after the CBTi was 1.06 (*p* = 0.883, 95% CI 0.52 to 2.15). At six weeks, the headache frequency reduced by 49% in the CBTi group and by 25% for controls. The odds ratio of a participant having a headache after CBTi was 0.40 (*p* = 0.028, 95% CI 0.17 to 0.91) in comparison to control. As they assessed two endpoints of their primary outcome, they used a criterion of *p* < 0.025 to determine significance, which their results did not meet. These analyses were based on total person days with/without headaches reported by participants rather than headache days per person. It is unclear how this was accounted for in the analyses. A 50% reduction in headache frequency at follow up was reported by 5/15 (33%) of control 7/16 (44%) of the CBTi group (no statistical test performed).

### Headache-related disability

#### Educational interventions

Underwood *et al.* (2023a) reported no significant effects of their educational and self-management support intervention at 12 months (*p* = 0.325, mean difference (−0.7, 95% CI −1.97 to 0.65)) using the Headache Impact Test (HIT-6, scale range 36–78) (Kosinski *et al.*, 2003). The limits of the 95% confidence interval exclude the target difference of 2.0 set for the trial.

#### Psychological interventions

Grazzi *et al.* (2023) reported improved outcomes on Migraine Specific Quality of Life Questionnaire (MSQ) and Migraine Disability Assessment Test (MIDAS) at 12 months only, with no difference at intermediate timepoints at any timepoint on HIT-6 and World Health Organisation Disability Assessment Schedule (WHODAS-12). These data are presented graphically only. They further report statistically significant difference in proportion with ≥6 point improvement in HIT-6; 29/78 (37%) of the control group and 45/76 (59%) of the intervention group [chi squared *p* = 0.006, odds ratio 2.45, 95% CI 1.28 to 4.69) had achieved the reduction at 12 months.

Seng *et al.* (2021) reported that the estimated proportion of people reporting severe disability (MIDAS score of ≥21) was reduced by 16.4% in participants receiving mindfulness-based cognitive therapy and increased by 8.4% in participants receiving usual care. The mean difference in MIDAS scores (0-270) was −0.7 (95% CI: −1.27, −0.08). The limits of the 95% confidence interval exclude the target difference of 1.29 set for the trial. Seng *et al.* (2021) also used the Headache Disability Inventory (Jacobson *et al.*, 1994) to measure headache disability, but no significant differences were found.

#### Behavioural interventions

Smitherman *et al.* (2016) reported no differences in MIDAS and HIT-6 scores at two weeks or six weeks after correcting for baseline scores. However, no analysis is presented.

### Pain intensity/severity of symptoms

#### Educational interventions

Rashid-Tavalai *et al*. (2015) used the Migraine Headache Index (Hamedanizadeh *et al.*, 2010) and reported that the intervention did not have a significant effect on pain severity (*p* = 0.26).

#### Psychological interventions

Seng *et al.* (2021) found that the average attack intensity over 30 days did not differ between groups at four months; difference −0.02 (95%CI −0.16, 0.13, scale range 0–3).

#### Behavioural interventions

Calhoun and Ford (2007) expressed pain intensity as a headache index score. They calculated it by multiplying the number of days with severe headache by 3, moderate by 2 and mild by 1. After summating the score, the authors reported a significant difference between groups (*p* = 0.001). Between-group difference is not presented.

Smitherman *et al.* (2016) determined headache severity by asking participants to rate each headache using a 0 to 10 scale. The authors stated that there were no differences between groups when controlling for baseline; however, no statistical analyses were presented.

### Quality of life

#### Educational interventions

Rashid-Tavalai *et al*. (2015) used the World Health Organisation Quality of Life (WHO-QoL) questionnaire (Saxena *et al.*, 1997). When comparing the scores between groups at seven weeks they reported a statistically significant difference (*p* = 0.049).

#### Psychological interventions

Grazzi *et al.* (2023) reported that the participants in the intervention group better quality of life scores at 12 months, but the results were not statistically significant.

### Psychological wellbeing

#### Behavioural interventions

Smitherman *et al.* (2016) reported no difference between CBTi and control groups at six weeks in Patient Health Questionnaire (PHQ-9) (Kroenke *et al.*, 2001) scores for depression (*p* = 0.054), but not in the Generalised Anxiety Disorder (GAD-7) (Spitzer *et al.*, 2006) scores for anxiety (*p* = 0.420).

### Medication consumption

#### Behavioural interventions

Calhoun and Ford (2007) reported that at baseline 74% of all participants overused medication; however, 12 weeks after the intervention this was stopped in all patients who reverted to episodic migraine (48%) and in 60% of participants that did not revert.

#### Psychological interventions

Grazzi *et al.* (2023) reported that the participants in the intervention group showed a significantly lower consumption of medications (including total drug intake and non-steroidal anti-inflammatory drugs) than the control group at 12 months, but not the triptans. The data was presented graphically only.

## Discussion

### Summary of the main findings

In this systematic review, we investigated the effectiveness of educational, behavioural, psychological, and self-management support interventions for chronic migraine. We analysed two educational, two psycho-educational and two behavioural trials. It is disappointing how few eligible studies we identified, with so few participants (*N* = 713), when chronic migraine is such a common and sometimes profoundly disabling condition. It is further disappointing that the published trials are not adequately powered to identify important benefits for people with chronic migraine. Two, Calhoun and Ford (2007) (*N* = 43) and Smitherman *et al.* (2016) (*N* = 31) are explicitly pilot studies; two, Seng *et al.* (2021) (*N* = 31) and Underwood *et al.* (2023a) (*N* = 396), only report on the sub-set of the overall trial population with chronic migraine; and one, Rashid-Tavalai *et al*. (2015) (*N* = 35), does not provide a priori sample size calculation. Only Grazzi *et al.* (2023, 2022) (*N* = 177) present a sample size calculation relevant to a chronic migraine population, based on showing an improvement on portion achieving a ≥50% reduction in headache days from 48% to 68%. By way of a benchmark for the size of trials needed to show difference in headache-related quality of life, a simple trial designed to show a 2.0 difference in HIT-6, the worthwhile difference set by Underwood (Patel *et al.*, 2020) with the observed baseline standard deviation of 5.5, from that trial, with 80% power at the 5% significance level requires data on 240 participants. It is even more disappointing that there is insufficient good-quality evidence of effectiveness for any of the treatment approaches to support their use outside of a research context.

Underwood *et al.* (2023a) included both people with chronic migraine and people with chronic tension-type headache and episodic migraine to assess the effectiveness of their educational intervention. Only the primary outcome of HIT-6 at 12 months is reported separately for people with chronic migraine. The limits of the 95% confidence interval for HIT-6 at one year excluded the pre-specified target difference, allowing them to conclude that the intervention tested was not effective on HIT-6. The authors’ overall conclusion for the main study, including people with episodic migraine, is that their data excludes the possibility that the intervention tested might be effective. There would seem to be little justification for any further evaluation of approach used by Underwood *et al.* (2023a). For the remaining trials where there is at least some evidence, with exception of Grazzi, the reporting of the interventions tested is inadequate to allow replication. To our knowledge, none have used the Template for Intervention Description and Replication (TIDieR) checklist (Hoffmann *et al.*, 2014).

Calhoun and Ford (2007) found a significantly reduced headache frequency and pain severity in the behavioural intervention group at six weeks when compared to controls. As sleep problems can exacerbate chronic migraine (Schwedt, 2014), facilitating sleep hygiene through behavioural modifications could effectively improve headache-related outcomes for individuals with chronic migraine. Smitherman *et al.* (2016) also reported reductions in the odds of experiencing a headache following behavioural CBTi, but no statistical significance between groups. With both behavioural interventions in this review being almost identical, it is possible that Smitherman *et al.* (2016) failed to reach significance as their participants had comorbid insomnia, and simple behavioural sleep instructions may not be sufficient to achieve a clinically meaningful change in this population. Lastly, Smitherman *et al.* (2016) reported borderline significance between groups for the reduction in depression scores at six weeks. Though not specific for chronic migraine, CBT was effective in reducing depression scores for individuals with migraine in a 2012 trial (*N* = 213) (Bromberg *et al.*, 2012). Given the frequent co-occurrence of depression in people with migraine (Yang *et al.*, 2016), managing negative thoughts could be a valuable tool in improving their mental health (Martin *et al.*, 2015). However, Smitherman *et al.* (2016) did not focus on the cognitive component of CBT, which may explain why they did not find significant differences in depression scores.

The studies classified as psychological assessed the effectiveness of mindfulness-based cognitive therapy (Seng *et al.*, 2021) and coping skills (Rashid-Tavalai *et al*., 2015). In line with a Cochrane review of psychological therapies for migraines (Sharpe *et al.*, 2019), Seng *et al.* (2021) reported no significant difference in headache frequency between groups at four months. Seng *et al.* (2021) also reported no significant improvements in the average intensity of pain, whereas a systematic review demonstrated that interventions with mindfulness components had small effects on pain intensity in participants with migraines (Probyn *et al.*, 2017). However, Seng *et al.* (2021) reported a statistically significant reduction in headache-related disability using MIDAS questionnaire. Their results highlight that headache frequency and headache-related disability are not co-dependent. This is supported by the Delphi consensus process, where the authors recommended that non-pharmacological interventions should prioritise headache-related disability over headache frequency (Luedtke *et al.*, 2020). This is because interventions, such as mindfulness-based therapy, target the ability to function with pain, not eliminate the occurrence of the disease (Cherkin, 2021).

### Completeness and applicability of the evidence

We used the International Classification of Headache Disorders 3^rd^ edition (ICHD-3) (Olesen *et al.*, 2003) criteria for chronic migraine, which requires individuals to have 15 headaches a month, with eight migraines for longer than three months. These strict criteria meant that very few studies could be included in our analysis. This led us to exclude nearly all the data from Underwood *et al.* (2023a) where they had identified a population using an epidemiological definition of chronic headache rather than ICHD-3. The authors justified the inclusion of this population by explaining that in practice they would have access to this intervention and are therefore a clinically relevant population. Additionally, we excluded a trial with an average of 12 headache days a month (Cousins *et al.*, 2015), but a longitudinal study with over a thousand participants with chronic migraine showed that nearly 75% of those with chronic migraine at baseline dropped below this diagnostic boundary at least once in 12 months (Serrano *et al.*, 2017). Furthermore, participants just below the required diagnostic threshold for chronic migraine were reported to have comparable levels of headache-related disability (Chalmer *et al.*, 2020). Identifying such individuals would ensure that appropriate treatment is given according to their needs.

The trials included in this study had a majority female population. This is due to the higher prevalence of chronic migraine in women in comparison to men; a systematic review of the global epidemiology of chronic migraine found the prevalence was 2.5–6.5 times higher in women (Natoli *et al.*, 2010). Consequently, our analysis is less applicable to males with chronic migraine. Furthermore, of the three studies that reported on ethnicity, 77%–81% of the population were white reducing the generalisability of this data. We only included trials of adults aged 18 or over.

Educational, behavioural, and psychological interventions could become tools to manage chronic migraine long-term; however, we cannot determine any long-term effects from these studies.

### Quality of the evidence

No trial had low risk of bias, with reasons listed in Supplementary Material 3. We found the presentation of results by Seng *et al.* (2021) to be difficult to interpret due to lack of clarification in table legends. Rashid-Tavalai *et al*. (2015) used a scale called ‘Migraine Headache Index’, which we were unable to access. Additionally, no raw data were reported, leaving us unable to determine how pain intensity had been calculated. This affected the credibility of the conclusions drawn by the authors. The use of an odds ratio to report headache frequency by Smitherman *et al.* (2016) to be a non-standard approach, as ‘headache days’ is a continuous variable. Additionally, migraine days have been shown to cluster; thus, the probability of having a migraine on any day is affected by the occurrence of migraines on previous days (Barra *et al.*, 2020). We also noted the lack of clarity in reporting the effect of intervention on headache frequency by Calhoun and Ford (2007); the analysis was stated as being between-groups, but the supplementary graph seemed to indicate a within-group analysis, comparing baseline with follow-up. We contacted the authors for clarification but had no success, leaving this ambiguity unresolved. Grazzi present the majority of their secondary analyses in a graphical form only, limiting their interpretation.

Some studies did not specify what they meant by ‘usual care’ for their control groups. For instance, Rashid-Tavalai described it as ‘pharmacotherapy’, whereas Seng defined it as ‘standard migraine care’. These two trials were carried out in different countries; therefore, ‘usual’ treatment may mean different pharmacological regimes and may include additional strategies, such as headache diaries. Further clarification would be beneficial to facilitate comparison. Smitherman and Calhoun & Ford used sham instructions in addition to pharmacological management for their control group. These included five-step instructions, such as performing acupuncture twice daily and scheduling a consistent meal time. Underwood referred the control group back for usual care from their general practitioner along with recommendations for appropriate drug treatment and relaxation compact disc. Grazzi provided the most detail regarding their control group, specifying that their ‘treatment as usual’ condition consisted of overused medication withdrawal, education on proper medication use and prescriptions of suitable pharmacological agents. They offered a comprehensive summary of the methods they utilised to facilitate withdrawal as well as the exact medication and dosage that was used as usual migraine prophylaxis. This offers clarity and facilitates replication in future studies.

### Practical implications

Three interventions utilised graduate-level psychologists to deliver the interventions. A 2017 systematic review found no evidence that psychological interventions delivered by a specialist were more effective than those delivered by an allied healthcare professional (Probyn *et al.*, 2017). This has important implications for the cost of delivering the intervention. In contrast, a feasibility study found that it was not practical to include lay migraineurs, as the unpredictability of migraine attacks prevented them from being able to commit to delivering the sessions (White *et al.*, 2019).

Whilst largely focused on drug trials, ICHD guideline for prophylactic treatment of chronic migraine is a useful tool to inform trial design (Silberstein, 2008). To reduce variability between trials in future, authors should adhere to the ICHD-3 criteria when recruiting the participants and clearly define their population. To facilitate reproducibility and critical appraisal, we encourage the use of the CONSORT checklist (Hopewell *et al.,* 2025). A published protocol, adhering to the SPIRIT guidelines would further enhance interpretation (Chan *et al*., 2025). Better descriptions of both control and intervention groups, including the underpinning theory informing the content of the active intervention is needed. This may be best addressed by publication of separate intervention development papers, as done by Underwood (Patel, 2019). We were unable to do a meta-analysis due to the heterogeneity of the reported outcomes. Future trials could utilise the two-domain core outcome, headache frequency and headache-related disability identified as part of a core outcome set. This will allow for meaningful pooling of data in future reviews.

### Strengths and limitations

We followed the PRISMA checklist for the reporting of this systematic review and prospectively registered our protocol. We assessed the trials using the Cochrane Risk of bias tool. We could not perform any meta-analyses to estimate the overall effect of the interventions due to the heterogeneity of the trials. Moreover, we only included articles published in English and may have missed relevant trials in other languages.

Our search was conducted in July 2023; therefore, we re-ran the original search in PubMed in June 2025 to identify any newly published trials. The updated search yielded 26 new studies; however, none met our predefined eligibility criteria. Therefore, our findings remain current and up to date.

## Conclusion

Chronic migraine is a disabling condition for which pharmacological treatment is not always sufficient. Therefore, non-pharmacological support interventions could complement the existing treatment and improve headache-related outcomes. We found some weak evidence for the effectiveness of educational, behavioural, and psychological interventions in reducing headache frequency, headache-related disability, and pain intensity. However, research by Underwood *et al.* (2023a) demonstrated that in a large randomised controlled trial, with long-term follow-up, educational/behavioural treatment for chronic migraine had no clinically meaningful improvement for patients. These interventions cannot yet be considered part of routine care. Nevertheless, patients ought to be actively involved in the decision-making process when determining the most appropriate management plan for chronic migraine, including how to make the best use of medication for migraine prophylaxis. Focus should also be placed on finding alternative treatments to better support individuals living with this debilitating condition.

## Supporting information

Hailston et al. supplementary material 1 Hailston et al. supplementary material

Hailston et al. supplementary material 2 Hailston et al. supplementary material

Hailston et al. supplementary material 3 Hailston et al. supplementary material

## Data Availability

Supplemental material for this article is available online (CRD42021260376).

## Supplementary material

The supplementary material for this article can be found at https://doi.org/10.1017/S1463423625100571
