# Supplementary material for: The effectiveness of educational, behavioural, and cognitive self-management support interventions for chronic migraine: a systematic review
Source: Prim Health Care Res Dev. 2025 Dec 3;26:e98. doi: 10.1017/S1463423625100571 (PMC12721982; doi:10.1017/S1463423625100571)
Supplement: Hailston et al. supplementary material 1 — Hailston et al. supplementary material [file S1463423625100571sup001.docx]

**Cochrane**

ID Search Hits

#1 MeSH descriptor: [Headache] explode all trees 2495

#2 MeSH descriptor: [Headache Disorders] explode all trees 3562

#3 MeSH descriptor: [Migraine Disorders] explode all trees 2831

#4 MeSH descriptor: [Migraine with Aura] explode all trees 130

#5 MeSH descriptor: [Headache Disorders, Primary] explode all trees 3206

#6 MeSH descriptor: [Migraine without Aura] explode all trees 151

#7 (headache):ti,ab,kw 32967

#8 ("migraine headache"):ti,ab,kw 1047

#9 headach* near/3 (stress or idiopathic or psychogenic or psychomyogenic or oridinary) 156

#10 chronic near/2 daily near/2 headache 114

#11 chronic near/2 migraine 1265

#12 chronic near/2 headache 704

#13 long term headache 3990

#14 (withdrawal or overuse or "over use" or "over-use" or misuse or "mis-use" or abuse or induced) near/5 (medication* or medicine* or analges* or drug* or opiate* or opioid* or NSAIDS or non-opiate* or non opiate* or ergot* or painkiller* or pain killer* or pain-killer*) near/5 (headache* or migraine*) 3510

#15 (rebound or transformed) near/5 (headache* or migraine*) 781

#16 MeSH descriptor: [Psychotherapy, Group] explode all trees 3588

#17 (group therapy):ti,ab,kw 347327

#18 MeSH descriptor: [Pain Management] explode all trees 4143

#19 MeSH descriptor: [Self-Management] explode all trees 589

#20 MeSH descriptor: [Self-Help Groups] explode all trees 788

#21 MeSH descriptor: [Self Care] explode all trees 5929

#22 ((self-management or self management or self-care)):ti,ab,kw 26975

#23 (training near/5 program*):ti,ab,kw 16497

#24 MeSH descriptor: [Cognitive Behavioral Therapy] explode all trees 9773

#25 ((behavioral or behavioural)):ti,ab,kw 58217

#26 MeSH descriptor: [Psychiatric Rehabilitation] explode all trees 45

#27 (psychosocial):ti,ab,kw 17138

#28 MeSH descriptor: [Patient Care Team] explode all trees 1771

#29 (randomized controlled trial):ti,ab,kw 635832

#30 (controlled clinical trial):ti,ab,kw 540411

#31 (trial):ti 352365

#32 (randomly):ab 270932

#33 (randomised):ab 714751

#34 ("animal experiment"):ti,ab,kw 2453

#35 #1 or #2 or #3 or #4 or #5 or #6 or #7 or #8 or #9 or #10 or #11 or #12 or #13 or #14 or #15 35647

#36 #16 or #17 or #18 or #19 or #20 or #21 or #22 or #23 or #24 or #25 or #26 or #27 or #28 428098

#37 #29 or #30 or #31 or #32 or #33 1122475

#38 #37 not #34 1120261

#39 #35 and #36 and #38 12403

**Embase**

1 exp headache/ or exp migraine/ or exp transformed migraine/ or exp chronic daily headache/ or exp primary headache/ 299390

2 exp migraine with aura/ or exp migraine without aura/ 6863

3 "headache and facial pain"/ 1923

4 (headache* or migraine*).mp. 353484

5 (headache* adj3 (psychomyogenic or stress-> physiological stress or ordinary or idiopathic or pychogenic)).tw. 419

6 ((chronic adj2 daily adj2 headache*) or (daily adj2 persistent adj2 headache*)).ti,ab. 1790

7 (chronic adj2 migrain*).mp. 6716

8 (chronic adj2 headach*).mp. 8214

9 (withdrawal or overuse or "over use" or "over-use" or misuse or "mis-use" or abuse or induced).mp. adj5 (medication* or medicine* or analges* or drug* or opiate* or opioid* or NSAIDS or non-opiate* or non opiate* or ergot* or triptan* or painkiller* or pain killer* or pain-killer*).ti,ab. adj5 (headache* or migraine*).ti,ab. 3122

10 ((rebound or transformed) adj5 (headache* or migraine*)).ti,ab. 513

11 exp group therapy/ 23016

12 group therapy.ti,ab. 7168

13 exp self help/ or exp self-help/ 14562

14 exp self care/ or exp self-care/ 91625

15 (self-management or self management or self-care or self care).ti,ab. 57292

16 (training adj5 program*).ti,ab. 81184

17 exp behavioral therapy/ 65190

18 exp cognitive therapy/ 63698

19 (cognitive therapy or CBT).ti,ab. 22282

20 exp psychosocial rehabilitation/ 1667

21 exp controlled clinical trial/ 875704

22 randomi?ed.ab. 934747

23 randomly.ab. 492780

24 trial.ti. 351192

25 1 or 2 or 3 or 4 or 5 or 6 or 7 or 8 or 9 or 10 353496

26 11 or 12 or 13 or 14 or 15 or 16 or 17 or 18 or 19 or 20 305039

27 21 or 22 or 23 or 24 1772509

28 25 and 26 and 27 1005

29 (exp animal/ or exp invertebrate/ or nonhuman/ or animal experiment/ or animal tissue/ or animal model/ or exp plant/ or exp fungus/) not (exp human/ or human tissue/) 8087385

30 28 not 29 1004

31 limit 30 to (article or article in press) 570

**Web of Science**

**headache* or migraine* or "chronic headache*" or "chronic migraine*" (Topic) and "cognitive behavio$ral therapy" or "cognitive therapy" or "pain management" or "group therapy" or "self management"or “self-management” or “self care” or “self-care” or “self help” or “self-help” or (training and program*) or “Interdisciplinary Treatment Approach” or “Multidisciplinary Treatment Approach” or “Integrated Care” or CBT (Topic) and RCT or "Randomi?ed control trial*" or Randomly or "control trials" or randomi?ed (Topic)**

**PsychINFO**

1 exp headache/ or exp headache disorders/ or exp migraine/ or exp migraine disorders/ 15807

2 (migraine adj5 aura).ti,ab. 2277

3 (headache* or migraine*).mp. 25098

4 (headache* adj3 (psychomyogenic or stress or ordinary or idiopathic or psychogenic)).tw. 342

5 ((chronic adj2 daily adj2 headache*) or (daily adj2 persistent adj2 headache*)).ti,ab. 517

6 (chronic adj2 migrain*).mp. 1227

7 (chronic adj2 headach*).mp. 1845

8 long term headache*.mp. 13

9 (withdrawal or overuse or "over use" or "over-use" or misuse or "mis-use" or abuse or induced).mp. adj5 (medication* or medicine* or analges* or drug* or opiate* or opioid* or NSAIDS or non-opiate* or non opiate* or ergot* or painkiller* or pain killer* or pain-killer* or triptan*).ti,ab. adj5 (headache* or migraine*).ti,ab. 779

10 ((rebound or transformed) adj5 (headache* or migraine*)).ti,ab. 173

11 1 or 2 or 3 or 4 or 5 or 6 or 7 or 8 or 9 or 10 25098

12 exp pain management/ 10296

13 exp group psychotherapy/ 23449

14 exp self management/ or exp self-help/ or exp self care/ or exp self-management/ 14416

15 (self-management or self management or self-care or self care).ti,ab. 19729

16 (training adj5 program*).ti,ab. 37700

17 exp behavior therapy/ 21789

18 exp cognitive therapy/ 13723

19 (cognitive therapy or CBT).ti,ab. 20564

20 exp psychosocial rehabilitation/ 13855

21 exp Group Psychotherapy/ 23449

22 exp Interdisciplinary Treatment Approach/ 7561

23 exp manual/ 3955

24 exp randomized controlled trials/ 1054

25 exp clinical trials/ 13018

26 randomi?ed.ab. 87274

27 randomly.ab. 77961

28 trial.ti. 34044

29 12 or 13 or 14 or 15 or 16 or 17 or 18 or 19 or 20 or 21 or 22 or 23 163377

30 24 or 25 or 26 or 27 or 28 172210

31 11 and 29 and 30 196

**Medline**

1 exp headache/ or exp headache disorders/ or exp migraine/ or exp migraine disorders/ 62066

2 exp migraine with aura/ or exp migraine without aura/ 2497

3 (headache* or migraine*).mp. 124182

4 (headache* adj3 (psychomyogenic or stress or ordinary or idiopathic or pychogenic)).tw. 515

5 ((chronic adj2 daily adj2 headache*) or (daily adj2 persistent adj2 headache*)).ti,ab. 1077

6 (chronic adj2 migrain*).mp. 2977

7 (chronic adj2 headach*).mp. 4152

8 long term headache*.mp. 28

9 (withdrawal or overuse or "over use" or "over-use" or misuse or "mis-use" or abuse or induced).mp. adj5 (medication* or medicine* or analges* or drug* or opiate* or opioid* or NSAIDS or non-opiate* or non opiate* or ergot* or painkiller* or pain killer* or pain-killer* or triptan*).ti,ab. adj5 (headache* or migraine*).ti,ab. 1770

10 ((rebound or transformed) adj5 (headache* or migraine*)).ti,ab. 358

11 1 or 2 or 3 or 4 or 5 or 6 or 7 or 8 or 9 or 10 124310

12 exp pain management/ 38099

13 exp group therapy/ 27408

14 group therapy.ti,ab. 4478

15 exp self management/ or exp self help/ or exp self care/ or exp self-help/ or exp self-care/ 61918

16 (self-management or self management or self-care or self care).ti,ab. 40083

17 (training adj5 program*).ti,ab. 58877

18 exp behavioral therapy/ 32626

19 exp cognitive behavioral therapy/ 32626

20 (cognitive therapy or CBT).ti,ab. 14881

21 exp psychosocial intervention/ or exp psychosocial support systems/ 1255

22 exp Psychotherapy, Group/ 27408

23 randomi?ed control* trial.pt. 550502

24 controlled clinical trial.pt. 94543

25 randomi?ed.ab. 646533

26 randomly.ab. 370002

27 trial.ti. 251278

28 12 or 13 or 14 or 15 or 16 or 17 or 18 or 19 or 20 or 21 or 22 240362

29 23 or 24 or 25 or 26 or 27 1264538

30 11 and 28 and 29 425

31 exp animals/ not humans.sh. 4916277

32 30 not 31 425

**Scopus**

 ( ( headache*  OR  migraine* )  AND  ( "cognitive behaviour* therap*"  OR  "cognitive behavior* therap*"  OR  "cognitive therap*"  OR  "pain management"  OR  "group therap*"  OR  "self-management"  OR  "self-management"  OR  "self care"  OR  "self-care"  OR  "self help"  OR  "self-help"  OR  "interdisciplinary treatment approach*"  OR  "multidisciplinary treatment approach*"  OR  "integrat* care"  OR  cbt )  AND  ( rct  OR  "randomi?ed control* trial*"  OR  randomly  OR  "control* trial*" ) )
