# Supplementary material for: The effectiveness of educational, behavioural, and cognitive self-management support interventions for chronic migraine: a systematic review
Source: Prim Health Care Res Dev. 2025 Dec 3;26:e98. doi: 10.1017/S1463423625100571 (PMC12721982; doi:10.1017/S1463423625100571)
Supplement: Hailston et al. supplementary material 2 — Hailston et al. supplementary material [file S1463423625100571sup002.docx]

Excluded studies after full-title screening, with reason

| **Reason** | **Number of studies excluded** | **Studies** |
| --- | --- | --- |
| Less than 15 participants per treatment arm with chronic migraine | 2 | Bond DS, Thomas JG, Lipton RB, Roth J, Pavlovic JM, Rathier L, O'Leary KC, Evans EW, Wing RR. Behavioral weight loss intervention for migraine: a randomized controlled trial. Obesity. 2018 Jan;26(1):81-7.  Martin PR, Reece J, Callan M, MacLeod C, Kaur A, Gregg K, Goadsby PJ. Behavioral management of the triggers of recurrent headache: a randomized controlled trial. Behaviour research and therapy. 2014 Oct 1;61:1-1. |
| No data on what type of migraine | 2 | Bhombal ST, Usman A, Ghufran M. Effectiveness of behavioural management on migraine in adult patients visiting family practice clinics: a randomized controlled trial. JPMA. 2014 Aug 15;64(900).  Mahmoudzadeh ZF, Raiesifar A, Ebadi A. The effect of orem’s self-care model on quality of life in patients with migraine: A randomized clinical trial. 2016. |
| Less than 75% participants with chronic migraine | 6 | Devineni T, Blanchard EB. A randomized controlled trial of an internet-based treatment for chronic headache. Behaviour research and therapy. 2005 Mar 1;43(3):277-92.  Fernando Prieto Peres M, Prieto Peres Mercante J, Belitardo de Oliveira A. Non‐Pharmacological Treatment for Primary Headaches Prevention and Lifestyle Changes in a Low‐Income Community of Brazil: A Randomized Clinical Trial. Headache: The Journal of Head and Face Pain. 2019 Jan;59(1):86-96.  Mo'tamedi H, Rezaiemaram P, Tavallaie A. The effectiveness of a group‐based acceptance and commitment additive therapy on rehabilitation of female outpatients with chronic headache: Preliminary findings reducing 3 dimensions of headache impact. Headache: The Journal of Head and Face Pain. 2012 Jul;52(7):1106-19.  Seng EK, Singer AB, Metts C, Grinberg AS, Patel ZS, Marzouk M, Rosenberg L, Day M, Minen MT, Lipton RB, Buse DC. Does mindfulness‐based cognitive therapy for migraine reduce migraine‐related disability in people with episodic and chronic migraine? A phase 2b pilot randomized clinical trial. Headache: The Journal of Head and Face Pain. 2019 Oct;59(9):1448-67.  Tavallaei V, Rezapour-Mirsaleh Y, Rezaiemaram P, Saadat SH. Mindfulness for female outpatients with chronic primary headaches: an internet-based bibliotherapy. European journal of translational myology. 2018 Apr 24;28(2).  Vasiliou VS, Karademas EC, Christou Y, Papacostas S, Karekla M. Acceptance and commitment therapy for primary headache sufferers: a randomized controlled trial of efficacy. The Journal of Pain. 2021 Feb 1;22(2):143-60. |
| Excludes participants with chronic migraines | 3 | Mérelle SY, Sorbi MJ, Van Doornen LJ, Passchier J. Migraine patients as trainers of their fellow patients in non-pharmacological preventive attack management: short-term effects of a randomized controlled trial. Cephalalgia. 2008 Feb;28(2):127-38.  Simshäuser K, Lüking M, Kaube H, Schultz C, Schmidt S. Is mindfulness-based stress reduction a promising and feasible intervention for patients suffering from migraine? A randomized controlled pilot trial. Complementary medicine research. 2020;27(1):19-30  Sorbi MJ, Kleiboer AM, Van Silfhout HG, Vink G, Passchier J. Medium-term effectiveness of online behavioral training in migraine self-management: A randomized trial controlled over 10 months. Cephalalgia. 2015 Jun;35(7):608-18. |
| Not random assignment | 2 | Grazzi L, Sansone E, Raggi A, D’Amico D, De Giorgio A, Leonardi M, De Torres L, Salgado-García F, Andrasik F. Mindfulness and pharmacological prophylaxis after withdrawal from medication overuse in patients with Chronic Migraine: an effectiveness trial with a one-year follow-up. The journal of headache and pain. 2017 Dec;18(1):1-2.  Kleiboer A, Sorbi M, Mérelle S, Passchier J, Doornen LV. Utility and preliminary effects of online digital assistance (ODA) for behavioral attack prevention in migraine. Telemedicine and e-Health. 2009 Sep 1;15(7):682-90. |
| Grey literature (dissertation) | 2 | Butler N. Mindfulness-Based Cognitive Therapy and Self-Efficacy in People with Migraine: Yeshiva University; 2021.  Singer AB. Mindfulness practice and perceived stress: a secondary analysis of a randomized controlled trial of mindfulness-based cognitive therapy for migraine: Yeshiva University; 2017. |
| Unclear how many patients had chronic migraine | 11 | Bakhshani NM, Amirani A, Amirifard H, Shahrakipoor M. The effectiveness of mindfulness-based stress reduction on perceived pain intensity and quality of life in patients with chronic headache. Global journal of health science. 2016 Apr;8(4):142.  Bromberg J, Wood ME, Black RA, Surette DA, Zacharoff KL, Chiauzzi EJ. A randomized trial of a web‐based intervention to improve migraine self‐management and coping. Headache: The Journal of Head and Face Pain. 2012 Feb;52(2):244-61.  Cousins S, Ridsdale L, Goldstein LH, Noble AJ, Moorey S, Seed P. A pilot study of cognitive behavioural therapy and relaxation for migraine headache: a randomised controlled trial. Journal of neurology. 2015 Dec;262(12):2764-72.  Day MA, Thorn BE, Ward LC, Rubin N, Hickman SD, Scogin F, Kilgo GR. Mindfulness-based cognitive therapy for the treatment of headache pain: a pilot study. The Clinical journal of pain. 2014 Feb 1;30(2):152-61.  Dindo LN, Recober A, Calarge CA, Zimmerman BM, Weinrib A, Marchman JN, Turvey C. One-day acceptance and commitment therapy compared to support for depressed migraine patients: A randomized clinical trial. Neurotherapeutics. 2020 Apr;17(2):743-53.  D'Souza PJ, Lumley MA, Kraft CA, Dooley JA. Relaxation training and written emotional disclosure for tension or migraine headaches: a randomized, controlled trial. Annals of Behavioral Medicine. 2008 Aug;36(1):21-32.  Kraft CA, Lumley MA, D'Souza PJ, Dooley JA. Emotional approach coping and self‐efficacy moderate the effects of written emotional disclosure and relaxation training for people with migraine headaches. British journal of health psychology. 2008 Feb;13(1):67-71.  Matchar DB, Harpole L, Samsa GP, Jurgelski A, Lipton RB, Silberstein SD, et al. The headache management trial: a randomized study of coordinated care. Headache: The Journal of Head and Face Pain. 2008;48(9):1294-310.  Rothrock JF, Parada VA, Sims C, Key K, Walters NS, Zweifler RM. The impact of intensive patient education on clinical outcome in a clinic‐based migraine population. Headache: The Journal of Head and Face Pain. 2006;46(5):726–31.  Shagbazyan AE, Kovalchuk NA, Tabeeva GR. Role of educational programs in management of patients with medication-overuse headache. Neurology, Neuropsychiatry, Psychosomatics. 2021 Jun 22;13(3):27-33.  Thorn BE, Pence LB, Ward LC, Kilgo G, Clements KL, Cross TH, Davis AM, Tsui PW. A randomized clinical trial of targeted cognitive behavioral treatment to reduce catastrophizing in chronic headache sufferers. The Journal of Pain. 2007 Dec 1;8(12):938-49. |
| Not relevant outcomes | 1 | Crawford MR, Luik AI, Espie CA, Taylor HL, Burgess HJ, Jones AL, Rush University Sleep Research Team, Ong JC. Digital cognitive behavioral therapy for insomnia in women with chronic migraines. Headache: the journal of head and face pain. 2020 May;60(5):902-15. |
| Conference journal abstract | 1 | Minen M, Adhikari S, Padikkala J, Goldberg E, Powers S, Tasneem S, Bagheri A, Lipton R. A Pilot Randomized Controlled Study of a Smartphone Delivered Progressive Muscle Relaxation Intervention for Migraine in Primary Care (623). |
