# Supplementary material for: The effectiveness of educational, behavioural, and cognitive self-management support interventions for chronic migraine: a systematic review
Source: Prim Health Care Res Dev. 2025 Dec 3;26:e98. doi: 10.1017/S1463423625100571 (PMC12721982; doi:10.1017/S1463423625100571)
Supplement: Hailston et al. supplementary material 3 — Hailston et al. supplementary material [file S1463423625100571sup003.docx]

Risk of Bias justification table

| **Bias Domain** | **Risk of bias judgement** | **Support for judgement** |
| --- | --- | --- |
| **Calhoun & Ford, 2007** | | |
| Domain 1: Risk of bias arising from the randomisation process | Some Concerns | No information on whether the allocation sequence was concealed until participants were enrolled on interventions |
| Domain 2: Risk of bias due to deviations from the intended interventions | Some Concerns | No reference to protocol |
| Domain 3: Missing outcome data | High | Intention to treat analysis not performed |
| Domain 4: Risk of bias in measurement of the outcome | Low | Participants self-reported results and were therefore the assessors. The participants were blinded |
| Domain 5: Risk of bias in selection of the reported result | Some concerns | No reference to protocol |
| **Rashid-Tavalai (2016)** | | |
| Domain 1: Risk of bias arising from the randomisation process | Some Concerns | Allocation process for randomisation not explained in sufficient detail |
| Domain 2: Risk of bias due to deviations from the intended interventions | Some concerns | Participants were not blinded, and there is no consideration or analysis of the expectations of the participants |
| Domain 3: Missing outcome data | High | 5 participants were excluded from analysis after randomisation |
| Domain 4: Risk of bias in measurement of the outcome | Some concerns | Participants were not blinded to the intervention and self-reported PROMS. |
| Domain 5: Risk of bias in selection of the reported result | Some concerns | No reference to trial protocol |
| **Grazzi (2022, 2023)** | | |
| Domain 1: Risk of bias arising from the randomisation process | Low | No concerns |
| Domain 2: Risk of bias due to deviations from the intended interventions | Some concerns | Participants were not blinded, and there is no consideration or analysis of the expectations of the participants |
| Domain 3: Missing outcome data | Some concerns | Participants lost to follow up were excluded from analysis, however, the reasons and proportions were similar between control and intervention group |
| Domain 4: Risk of bias in measurement of the outcome | Some concerns | Participants self-reported results and were therefore the assessors. How this was reported is not described. Participants were not blinded to the intervention and self-reported PROMs. |
| Domain 5: Risk of bias in selection of the reported result | Some Concerns | Trial protocol was published but did not include a pre-specified analysis plan |
| **Seng (2021)** | | |
| Domain 1: Risk of bias arising from the randomisation process | Some Concerns | No information comparing baseline characteristics between control and intervention group. |
| Domain 2: Risk of bias due to deviations from the intended interventions | Some concerns | Participants were not blinded, and there is no consideration or analysis of the expectations of the participants |
| Domain 3: Missing outcome data | Low | No concerns |
| Domain 4: Risk of bias in measurement of the outcome | Some concerns | Participants were not blinded to the intervention and self-reported PROMS |
| Domain 5: Risk of bias in selection of the reported result | Some Concerns | Trial protocol was published but did not include a pre-specified analysis plan |
| **Smitherman (2016)** | | |
| Domain 1: Risk of bias arising from the randomisation process | Low | No concerns |
| Domain 2: Risk of bias due to deviations from the intended interventions | Low | No concerns |
| Domain 3: Missing outcome data | Low | No concerns |
| Domain 4: Risk of bias in measurement of the outcome | Low | No concerns |
| Domain 5: Risk of bias in selection of the reported result | Some Concerns | Trial protocol was published but did not include a pre-specified analysis plan |
| **Underwood (2023)** | | |
| Domain 1: Risk of bias arising from the randomisation process | Low | No concerns |
| Domain 2: Risk of bias due to deviations from the intended interventions | Some concerns | Participants were not blinded, and there is no consideration or analysis of the expectations of the participants |
| Domain 3: Missing outcome data | Low | No concerns |
| Domain 4: Risk of bias in measurement of the outcome | Some concerns | Participants self-reported results and were therefore the assessors. Participants were not blinded to the intervention and self-reported PROMS |
| Domain 5: Risk of bias in selection of the reported result | Low | No concerns |
